# Supplementary material for: Dioctyl Phthalate-Modified Graphene Nanoplatelets: An Effective Additive for Enhanced Mechanical Properties of Natural Rubber
Source: Polymers (Basel). 2022 Jun 22;14(13):2541. doi: 10.3390/polym14132541 (PMC9269448; doi:10.3390/polym14132541)
Supplement: Supplementary file 1 [file polymers-14-02541-s001.zip › polymers-1738022-supplementary.pdf]

Dioctyl phthalate-modified graphene nanoplatelets: an effective additive for enhanced mechanical properties of natural rubber

Linh Nguyen Pham Duy[1], Chuong Bui[1], Liem Thanh Nguyen[1], Tung Huy Nguyen[1], Nguyen Thanh Tung[2], Duong Duc La[3,*]

[1]*Center for Polymer Composite and Paper, School of Chemical Engineering, Hanoi university of science and technology, Hai Ba Trung, Ha Noi, Vietnam*
[2] *Institute of Materials Science, Vietnam Academy of Science and Technology, 18-Hoang Quoc Viet, Hanoi, Vietnam*

[3]*Institute of Chemistry and Materials, Hoang Sam, Nghia Do, Cau Giay, Hanoi, Vietnam*

Keywords: graphene nanoplatelets; modified graphene, rubber; dioctyl phthalate; nanocomposite

Supporting Information

Herein is provided further information about the XRD patterns of dioctyl phthalate (DOP) and graphene nanoplatelets (GNPs), the stress-strain curve of GNPs/NR composite, comparative mechanical properties of the GNPs/NR with addition of pristine GNPs and modified GNPs. This material is available free of charge via the internet.

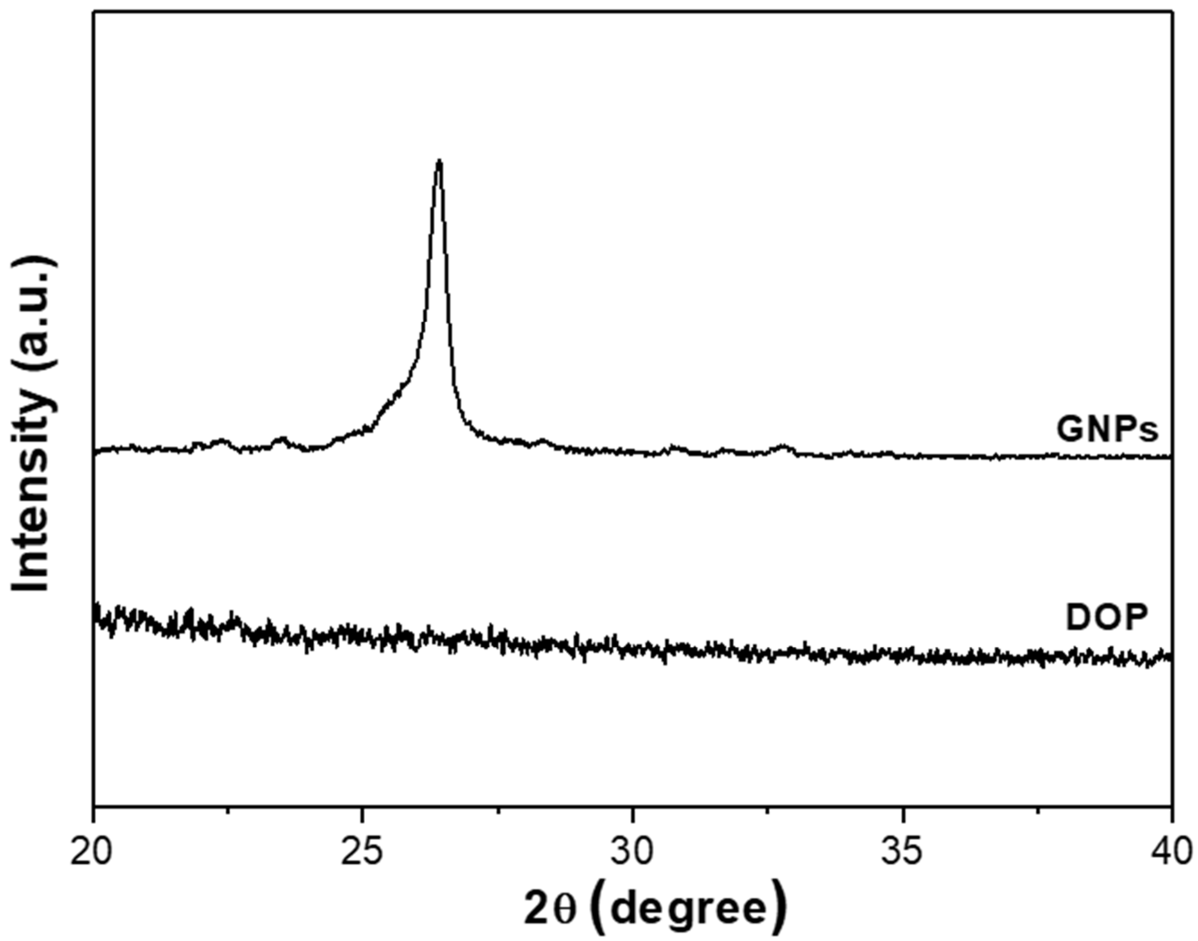

Figure S1. XRD patterns of dioctyl phthalate (DOP) and graphene nanoplatelets (GNPs).

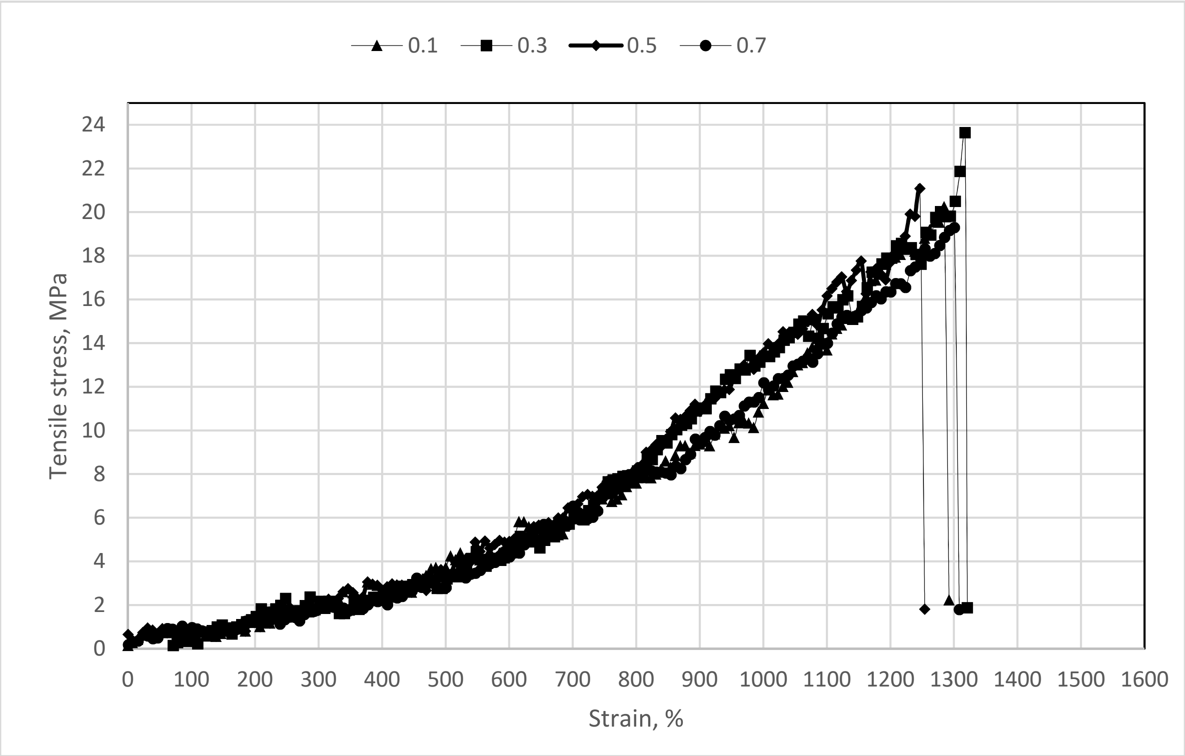

Figure S2. The stress-strain curve of GNPs/NR with GNPs loadings of 0.1, 0.3, 0.5, and 0.7 phr.

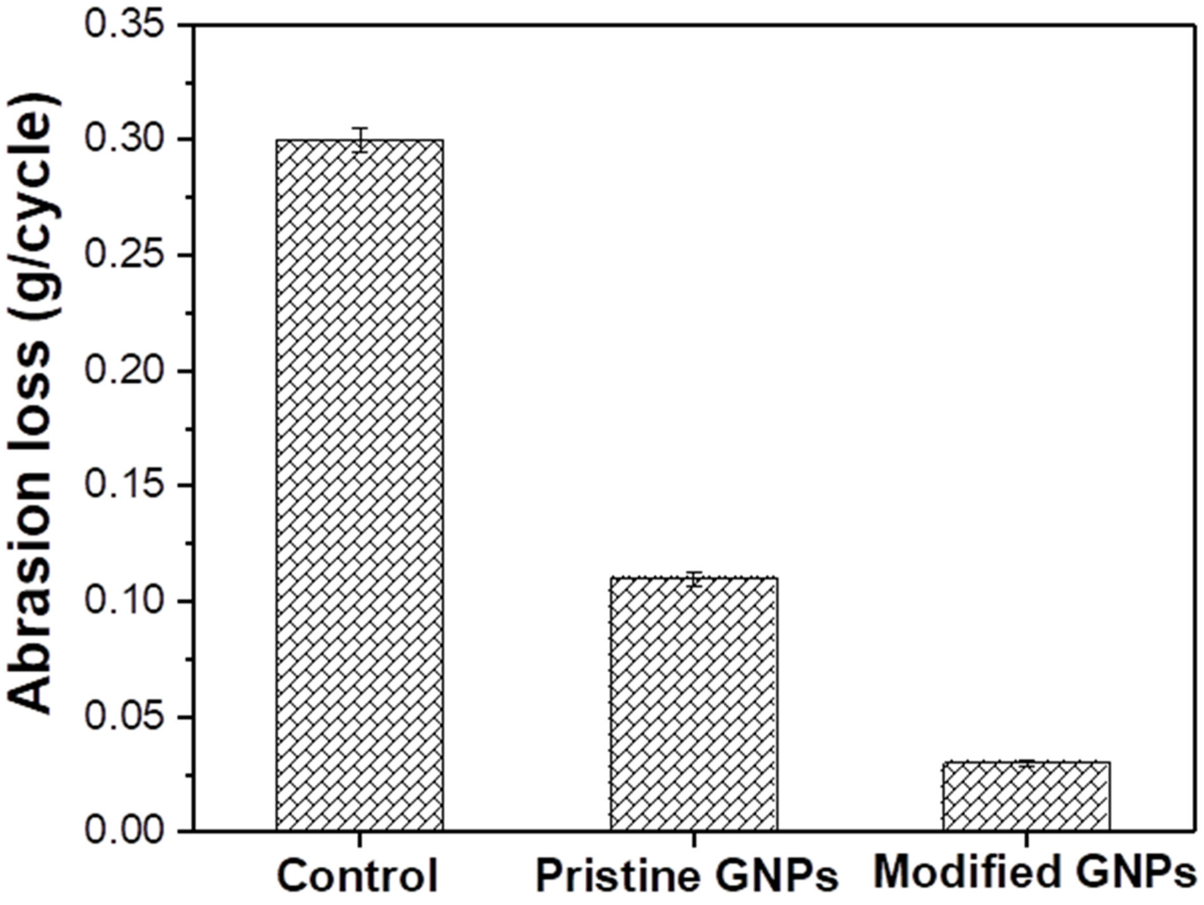

Figure S3. Comparison in abrasion loss of control, pristine GNPs, and modified GNPs at the loadings of 0.3 phr.

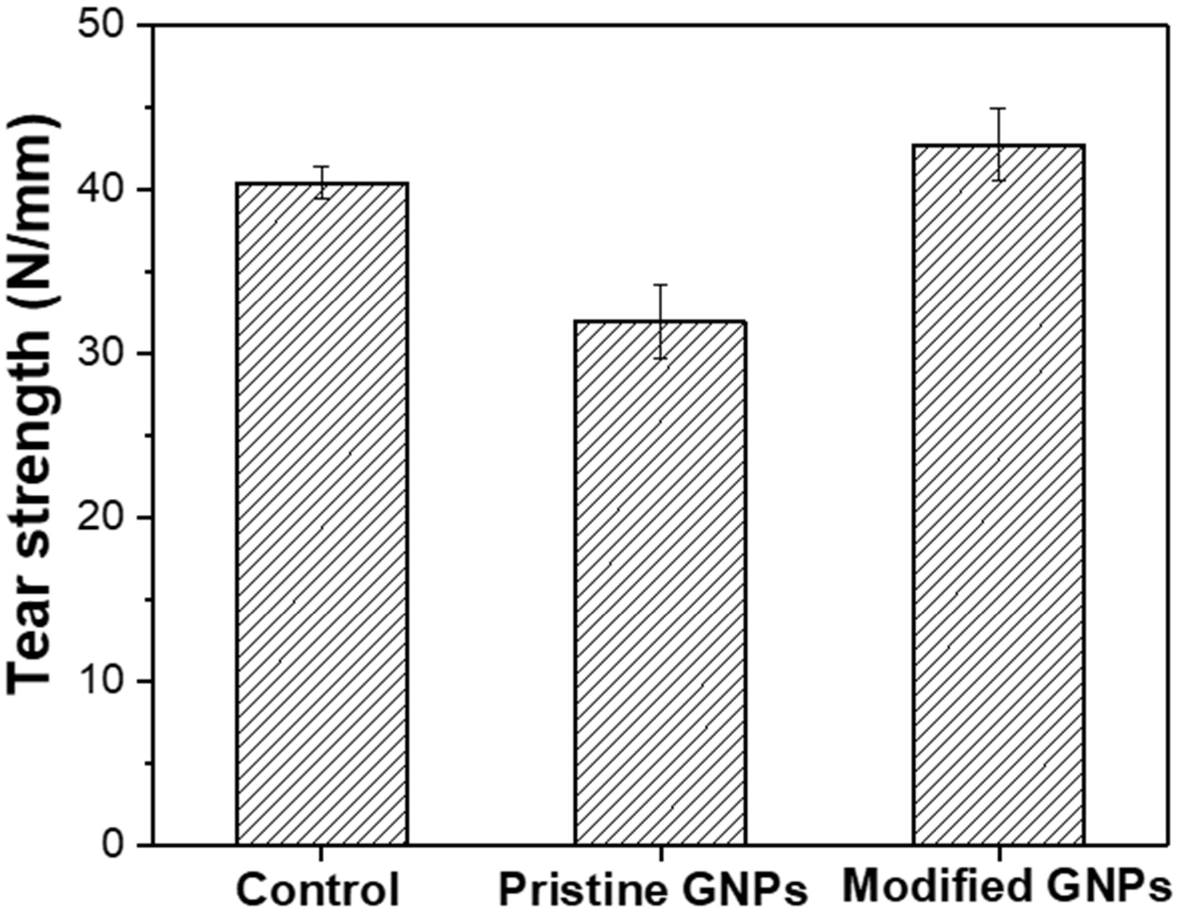

Figure S4. Comparison in tear strength of control, pristine GNPs, and modified GNPs at the loadings of 0.3 phr.

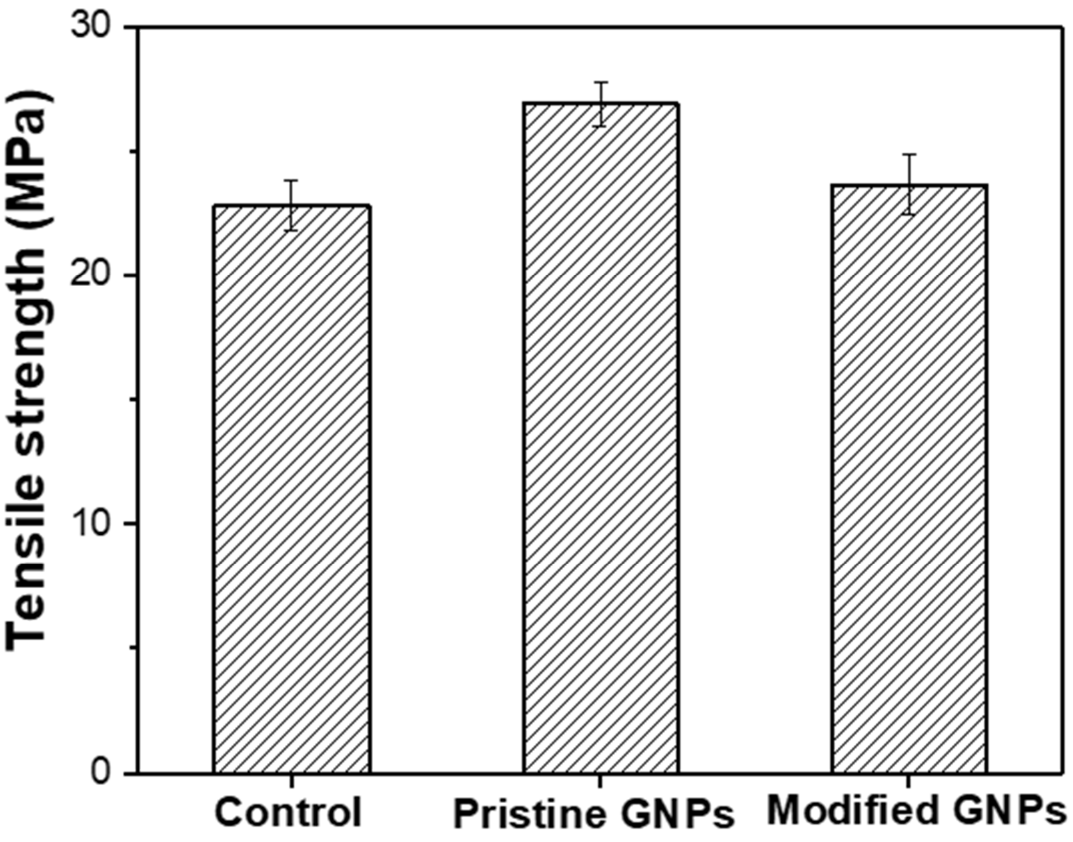

Figure S5. Comparison in tensile strength of control, pristine GNPs, and modified GNPs at the loadings of 0.3 phr.

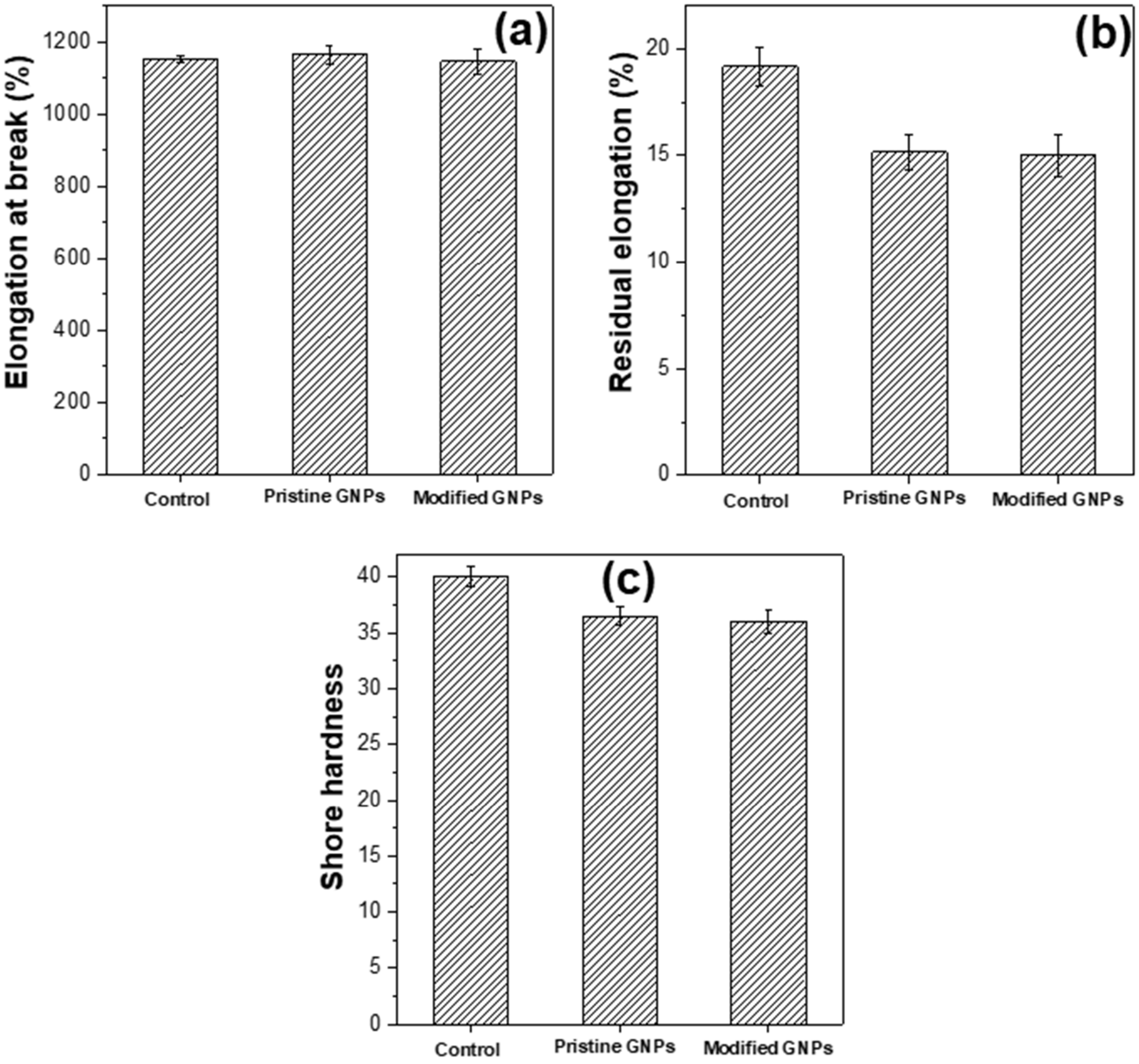

Figure S6. Comparison in (a) elongation at break, (b) residual elongation, and (b) Shore hardness of control, pristine GNPs, and modified GNPs at the loadings of 0.3 phr.
